# Protocol for EpiCom: A phase 3b/4 study of behavioral outcomes following adjunctive cannabidiol for the management of tuberous sclerosis complex-associated neuropsychiatric disorders (TAND)

**DOI:** 10.1371/journal.pone.0324648

**Published:** 2025-06-12

**Authors:** Agnies M. van Eeghen, Elizabeth A. Thiele, Sam Amin, Debopam Samanta, Anna C. Jansen, Joanne Stevens, Lisa Moore-Ramdin, Petrus J. de Vries

**Affiliations:** 1 Emma Center for Personalized Medicine, Emma Children’s Hospital, Amsterdam University Medical Centers, Amsterdam, Netherlands; 2 Advisium, ‘s Heeren Loo, Amersfoort, Netherlands; 3 Massachusetts General Hospital, Boston, Massachusetts, United States of America; 4 Paediatric Neurology, University Hospitals Bristol and Weston, Bristol, United Kingdom; 5 Child Neurology Section, Department of Pediatrics, University of Arkansas for Medical Sciences, Little Rock, Arkansas, United States of America; 6 Department of Pediatrics, Pediatric Neurology Unit, Antwerp University Hospital, Edegem, Belgium; 7 Jazz Pharmaceuticals, Inc., Philadelphia, Pennsylvania, United States of America; 8 Jazz Pharmaceuticals, Inc., London, United Kingdom; 9 Center for Autism Research in Africa, Division of Child & Adolescent Psychiatry, University of Cape Town, Cape Town, South Africa; Seirei Hamamatsu Hospital: Seirei Hamamatsu Byoin, JAPAN

## Abstract

Tuberous sclerosis complex (TSC)-associated neuropsychiatric disorders (TAND) affect ≈90% of individuals with TSC and significantly reduce their quality of life (QoL). However, there are limited studies assessing pharmacotherapy for TAND. A plant-derived highly purified pharmaceutical formulation of cannabidiol (CBD; Epidiolex^®^/Epidyolex^®^ oral solution) is approved for seizures associated with TSC. Anecdotal evidence also suggests psychiatric, neuropsychological, and behavioral benefits of CBD. EpiCom (**Epi**lepsy **Com**orbidities; NCT05864846; EU-CT, 2023-507426-17), a multicenter, open-label, phase 3b/4 study, with hybrid decentralized approach, was designed in collaboration with patient advisory groups and healthcare professionals to evaluate behavioral and other outcomes following adjunctive CBD treatment in individuals with TSC-associated seizures. EpiCom will enroll participants, aged 1–65 years (United States [US]) or 2–65 years (United Kingdom [UK], Canada, and Poland), who are starting CBD for seizures and have moderate/severe behavioral challenges according to the Caregiver Global Impression of Severity scale at screening. Participants will receive CBD (up to 25 mg/kg/d based on individual response and tolerability) in addition to their standard of care (SoC) for 26 weeks, after which participants may choose to continue CBD with SoC or SoC alone for an additional 26 weeks. Key efficacy endpoints include change from baseline on the Aberrant Behavior Checklist (e.g., irritability subscale) and the most problematic behavior on the TAND-Self-Report, Quantified Checklist. Changes in executive function, sleep, QoL, family functioning, seizure outcomes (severity, responder rates, seizure-free days), retention rate, and safety will be evaluated. The trial will enroll ≈75 participants at ≈20 sites across the US, the UK, Canada, and Poland. EpiCom will assess the changes in behavioral and other outcomes associated with TAND and seizure outcomes, including overall symptom severity and treatment retention, following adjunctive CBD treatment in individuals with TSC-associated seizures. The results will inform future studies evaluating pharmacotherapy for behavioral outcomes in similar populations.

## Introduction

Tuberous sclerosis complex (TSC) is a rare genetic disorder that is associated with pathogenic *TSC1* or *TSC2* gene variants, identified in 75%–90% of individuals with TSC, that have been shown to cause upregulation of the mechanistic target of rapamycin (mTOR), which in turn leads to formation of hamartomas in multiple organ systems, including the brain, heart, skin, eyes, kidneys, lungs, and liver [1,2]. Epilepsy is one of the most common neurologic manifestations of TSC, which affects approximately 85% of people with TSC [3]. In addition to seizures, about 90% of people with TSC experience TSC-associated neuropsychiatric disorders (TAND), which include a wide range of behavioral, psychiatric, intellectual, academic, neuropsychologic, and psychosocial manifestations [4–6]. Dysregulation of the mTOR signaling pathway in TSC may contribute to epileptogenesis and neuropsychiatric phenotypes [1,7,8]. Brain abnormalities and epilepsy in individuals with TSC are also implicated in the development of neuropsychiatric problems; coexistence of epilepsy at an early age has been shown to increase the risk of TAND, but neither structural brain abnormalities nor seizures are necessary or sufficient to lead to TAND [9–12].

The “burden of illness” associated with TAND has been identified by families as the greatest clinical burden of TSC, impacting quality of life (QoL) for individuals and their families. Adverse effects on individuals’ QoL include poor sleep, social stigma, social isolation, and depression, with families experiencing excessive financial and healthcare costs, caregiver stress, and employment difficulties [4,6,13]. There are limited treatment options for TAND, with only a handful of studies evaluating the effect of pharmacological or nonpharmacological interventions [4]. Thus, there is a significant need for more evidence-based treatment options for TAND [4,6].

The plant-derived pharmaceutical formulation of highly purified cannabidiol (CBD) is approved as Epidiolex^®^ in the United States (US) for the treatment of seizures associated with Lennox-Gastaut syndrome (LGS), Dravet syndrome (DS), and TSC in patients aged ≥1 year, and as Epidyolex^®^ in the European Union and the United Kingdom (UK) for the treatment of seizures associated with LGS and DS in conjunction with clobazam in patients aged ≥2 years, and as an adjunctive therapy for seizures associated with TSC in patients aged ≥2 years [14,15]. At present, there are limited data that CBD or any other antiseizure medication (ASM) can ameliorate aspects of TAND. However, results from a small cohort of 18 patients with TSC treated under the CBD Expanded Access Program (EAP) [16] and anecdotal reports from the TSC community of patients, caregivers, and healthcare professionals (HCPs) have suggested benefits with CBD treatment in behavioral (e.g., calm or relaxed behavior) and neuropsychological symptoms (e.g., increased attention span, awareness, and concentration) [17]. In the CBD EAP, in addition to reduced seizure frequency after 3 months of adjunctive CBD treatment, cognitive gains such as improvements in alertness, verbal communication, vocalization, and cognitive ability, and initiation of emotional and physical connections were reported in 12 of 14 individuals with parent- and physician-observed global developmental delays [16]. Additionally, behavioral improvements were observed in six of nine individuals with behavioral problems [16]. Behavioral and neuropsychological improvements were noted in individuals who had a reduction in seizure frequency as well as those who did not experience seizure reduction [16]. Thus, although the study was limited by a small sample size, these preliminary results suggest that CBD could be beneficial for the treatment of TAND.

TAND intervention evaluation studies, such as that for CBD, are complicated by the scarcity of instruments available for quantification of behavioral changes associated with TAND and are largely limited to generic instruments, which have limited validation, acceptance, or relevance for TAND [4,6]. Additionally, the TSC population is highly heterogeneous; therefore, the effect of the disorder and the treatments can be highly specific to an individual [5,6,18]. Efforts are underway to develop reliable tools that can be used to specifically evaluate TAND in the real-world setting and in clinical trials. The TAND Self-Report, Quantified Checklist (TAND-SQ), is one such tool that was developed and tested for feasibility and accessibility [19,20] and is currently being validated [21]. It is a quantifiable checklist that can be used by caregivers or individuals with TSC to assess TAND-related challenges as well as an individual’s TAND cluster profile, which includes autism-like behavior, dysregulated behavior, and eat/sleep, mood/anxiety, neuropsychological, overactive/impulsive, and scholastic clusters [6,22]. Identification of TAND clusters can help individuals with TSC and their care providers to plan for further evaluations, treatment, and support [19]. The TSC patient-reported outcome measure (TSC-PROM) for adults was recently evaluated and covers physical and mental functions, activity and participation, and social support aspects of TSC [23].

Given the rarity, complexity, and heterogeneity of TSC and TAND, and vulnerability of affected individuals as well as their caregivers, there is a greater impetus for collaborations among patients, caregivers, and HCPs in clinical trial design to drive interventions with measurable outcomes that are of value to the entire community [4,6,24]. Here we describe the study design of EpiCom (**Epi**lepsy **Com**orbidities; NCT05864846), an interventional, multicenter, open-label, single-arm, phase 3b/4 study, which was designed in collaboration with patient advisory groups (PAGs) and HCPs to evaluate behavioral and other co-occurring outcomes following treatment with adjunctive CBD in individuals with TSC-associated seizures. A primary consideration in designing the study was to evaluate endpoints that provide information on outcomes that can be evaluated in future in-depth clinical trials and are also meaningful to individuals with TSC and their families.

## Materials and methods

**Fig 1:**
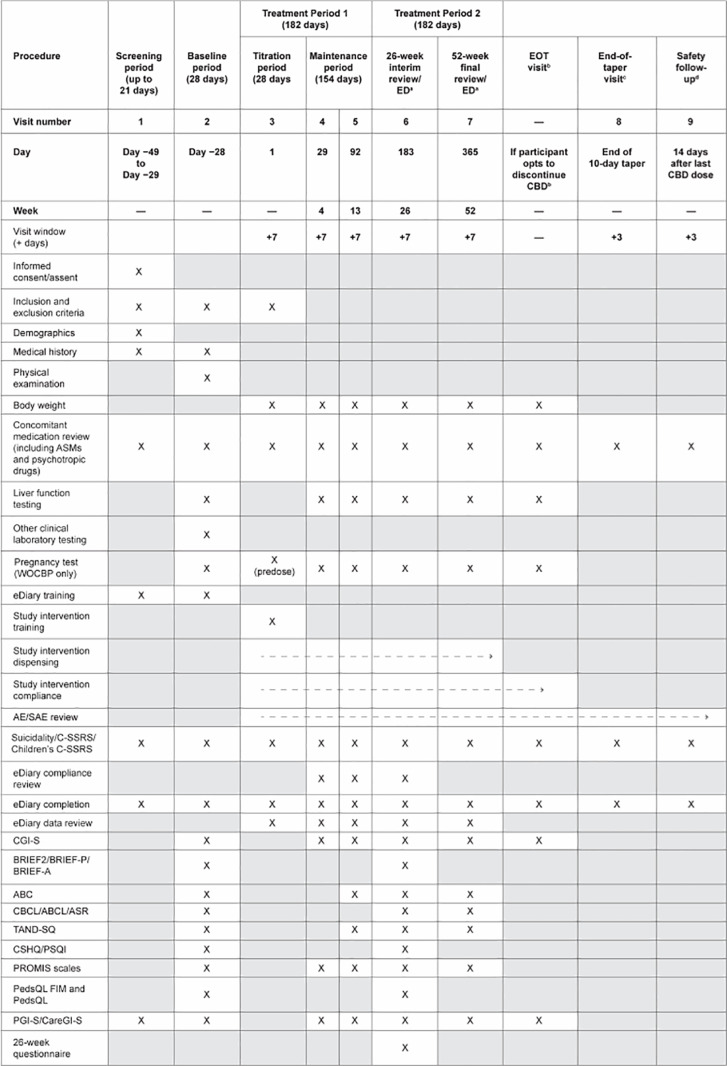
Schedule of assessments. ^a^The ED visit is only for participants who wish to discontinue the study and the study intervention (CBD-OS). These participants should initiate a 10-day tapering off of CBD-OS and then complete an end-of-taper visit, followed by a safety follow-up phone call. Participants who withdraw from the study before week 26 should complete assessments scheduled for the 26-week interim review visit, and participants who withdraw from the study after week 26 but before week 52 should complete assessments scheduled for the 52-week final review visit. ^b^Only for participants who wish to discontinue CBD-OS before the end of Treatment Period 1 or Treatment Period 2 but remain on study. These participants should initiate a 10-day tapering off of CBD-OS and then complete an end-of-taper visit, followed by a safety follow-up phone call. For participants who wish to remain on CBD-OS after the study, the 52-week final review visit is the last study visit. ^c^Only for participants who discontinue CBD-OS (after the 26-week interim review visit, 52-week final review visit, EOT visit, or ED visit). ^d^May be a phone call. ABC: Aberrant Behavior Checklist; ABCL: Adult Behavior Checklist; AE: adverse event; ASM: antiseizure medication; ASR: Adult Self Report; BRIEF-2: Behavior Rating Inventory of Executive Function-2; BRIEF-A: Behavior Rating Inventory of Executive Function for Adults; BRIEF-P: Behavior Rating Inventory of Executive Function Preschool; CareGI-S: Caregiver Global Impression of Severity; CBCL: Child Behavior Checklist; CBD-OS: cannabidiol oral solution; CGI-S: Clinician Global Impression of Severity; CSHQ: Children’s Sleep Habits Questionnaire; C-SSRS: Columbia–Suicide Severity Rating Scale; ED: early discontinuation; EOT: end of treatment; PedsQL: Pediatric Quality of Life Inventory; PedsQL FIM: Pediatric Quality of Life Survey Family Impact Module; PGI-S: Patient Global Impression of Severity; PROMIS: Patient-Reported Outcomes Measurement Information System; PSQI: Pittsburgh Sleep Quality Index; SAE: serious adverse event; TAND-SQ: Tuberous Sclerosis Complex-Associated Neuropsychiatric Disorders Self-report Quantified Checklist; WOCBP: women of childbearing potential.

EpiCom is an exploratory study that will assess the changes in various outcomes associated with TAND and seizure outcomes, including overall symptom severity and treatment retention, after starting adjunctive CBD treatment in individuals with TSC who experience seizures. Safety outcomes will also be evaluated. The schedule of assessments is presented in **Fig 1**.

### Co-designing approach for protocol development

**Fig 2:**
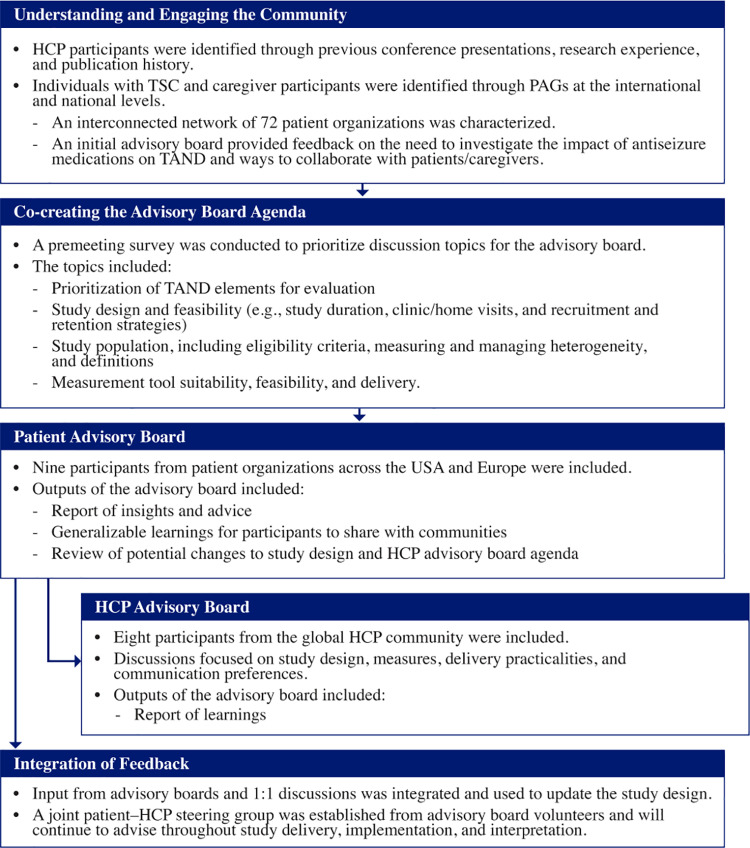
The process used to co-design the study with the TSC community. HCP: healthcare professional; PAGs: patient advisory groups; TAND: TSC-associated neuropsychiatric disorders; TSC: tuberous sclerosis complex; USA: United States of America.

The protocol for EpiCom trial was developed in collaboration with the TSC community (**Fig 2**). The process began by identifying HCPs through conference activities, publications, and clinical trials and finding patient and caregiver participants through PAGs at national and international levels. An initial advisory board with PAGs provided feedback on the need to investigate the impact of ASMs on TAND and ways to collaborate with patients and caregivers. Through premeeting surveys, patients and caregivers helped to co-create advisory board topics, including prioritization of TAND elements for evaluation, study design and feasibility, study population, and measurement tool suitability and feasibility. Feedback from the patient/caregiver advisory board was incorporated into the HCP advisory board. Insights (S1 Table) from both advisory boards were then used to design the study. The main aspects of the study design, including the effectiveness endpoints and timing of visits, were created to ensure that the study would measure clinically meaningful outcomes while minimizing participant/caregiver burden. A joint patient-HCP steering group was established and will advise throughout the study period. Input from nine patient organizations from the US and Europe and eight global HCPs was used to design the study protocol.

### Study design

**Fig 3:**
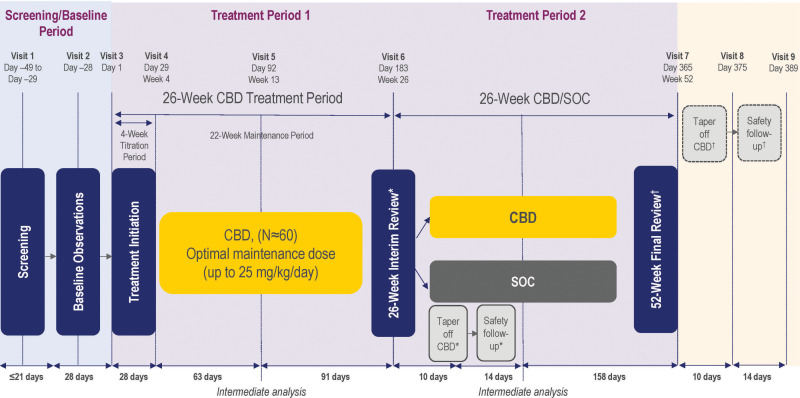
Study schema. *Participants who decide to discontinue CBD after the 26-week interim review visit but remain on study will form the SoC treatment arm. These participants will taper off CBD and complete a safety follow-up. ^†^Participants who decide to discontinue CBD after the 52-week final review visit will taper off CBD and complete a safety follow-up. For participants who wish to remain on CBD after the study, the 52-week final review visit is the last study visit. CBD: cannabidiol; SoC: standard of care.

The study consists of three phases (**Fig 3**): screening and baseline period (up to 49 days), 26-week treatment period 1 (adjunctive CBD treatment), and 26-week treatment period 2 (adjunctive CBD or standard of care [SoC]). Each participant will be enrolled in the study for up to 62 weeks, and the overall treatment duration is up to 54 weeks (including an option to taper off CBD at the end of the study if the participant would like to discontinue treatment). At screening, the prospective study participants or their caregivers will sign the informed consent form, followed by eligibility screening conducted up to a 21-day period. During this time, participants/caregivers will also receive training on how to record information in eDiaries. During the 4-week baseline period, participants will complete additional screening and baseline assessments and will be evaluated again for study eligibility.

During the first 26 weeks of treatment, participants will receive open-label CBD treatment in addition to the SoC. This treatment period consists of the following two phases: a 4-week titration period, during which the CBD dose will be titrated up to the participant’s optimal maximum dose (study site staff will train the participants or caregivers on how to dose and titrate CBD), and a 22-week maintenance period, during which the participants will maintain their optimal dose of CBD. Following a review after the first 26 weeks of treatment, participants will have the option to either continue adjunctive CBD treatment for another 26 weeks or discontinue CBD while maintaining their SoC treatment. Participants who choose to discontinue CBD after a 26-week interim review visit will be gradually withdrawn over a 10-day taper period and complete an end-of-taper visit and a follow-up safety assessment.

Participants who choose to continue treatment with CBD will maintain their dose for the remainder of treatment period 2, until the 52-week final review visit, which will also be the last study visit. Participants who do not continue CBD (outside of the study) after the 52-week final visit will undergo a 10-day taper period and complete an end-of-taper visit and a follow-up safety assessment. Participants will have the option to complete all study visits either at the study site or virtually (e.g., via telehealth or videoconference).

### Study population

Participants, aged 1–65 years in the US or 2–65 years outside the US, will be eligible to enroll if they fulfill the following key inclusion criteria: have a confirmed clinical diagnosis of TSC with a history of seizures in accordance with the 2012 International Tuberous Sclerosis Complex Consensus Conference criteria, which were updated in 2021 [13,25] and have behavioral problems—such as aggression, impulsivity, temper tantrum, self-injury, hyperactivity, extreme shyness, mood swings, poor eye contact, repetitive behaviors, restlessness, difficulty getting along with peers, and rigidity/inflexibility to procedure and/or change—that are considered moderate or severe according to the Caregiver Global Impression of Severity (CareGI-S) at screening. At baseline, prospective participants must have a most problematic behavior score of ≥6 on TAND-SQ to remain eligible. EpiCom participants must either be naive to CBD treatment or must have been off CBD treatment for ≥3 months before screening and taking ≥1 ASM at a stable dose. Concomitant ASMs are allowed, but any modifications or additions are to be avoided in the first 12 weeks, except changes permitted for participant safety reasons.

Prospective participants will be excluded from the study if they had any medical condition that in the investigator’s opinion could affect the study outcomes. This includes significant surgery for epilepsy that in the opinion of the investigator may impact the outcome’s assessment. In addition, participants will be excluded if they have initiation of felbamate within the year before screening, use recreational or medicinal cannabis or synthetic cannabinoid-based medications within the 3 months before screening and are unwilling to undergo a 1-month washout period before rescreening, have significant hepatic impairment, or have any history of suicidal behavior or any suicidal ideation.

### Treatment

All patients will receive adjunctive CBD (Epidiolex^®^/Epidyolex^®^, 100 mg/mL oral solution) treatment at a starting dose of 2.5 mg/kg administered twice daily during the first week of the titration period. The dose will be increased to 10 mg/kg/d during the second week and may be increased further in weekly increments of 2.5 mg/kg, depending on each participant’s clinical response and tolerability, to a maximum dose of 25 mg/kg/d. The dose may be reduced at any time in case of intolerance to treatment; otherwise, a stable dosage will be maintained throughout the study.

### Study endpoints, procedures, and assessments

Participating caregivers are expected to complete most assessments. Adult participants or caregivers will record all participant/caregiver-reported assessments in an eDiary throughout the study; seizures, including episodes of status epilepticus; changes in participants’ health and concomitant medications, including the use of rescue medications; and inpatient hospitalization for epilepsy should be recorded. Participants and/or caregivers will be trained on how to record information (including the CareGI-S at screening) in the eDiary. The effectiveness evaluation will include both TAND-SQ and seizure-related endpoints (**Table 1**).

**Table 1:** Study assessments and endpoints.

Assessments/instruments	Endpoint	Description of instruments	
		Validation status	Time to complete the assessment
**TAND Outcomes**			
ABC [26,27] A caregiver-reported tool that allows for assessments of problematic behavior in children and adults with developmental disabilities Caregivers will rate the participant’s specific symptoms across the following subscales: irritability, social withdrawal, stereotypic behavior, hyperactive noncompliance, and inappropriate speech	Change from baseline at weeks 13, 26, and 52	Can be used for children and adults Used for assessment in diseases with behavioral problems, including ASD, Down syndrome, and fragile X syndrome [26,28,29] Widely used for evaluating treatment outcomes in autism [30,31,32]	10–15 min
TAND-SQ [19,20] A checklist completed by caregivers or adults^a^ with TSC for assessment of symptoms across TAND-related domains (i.e., neuropsychiatric, cognitive, and behavioral symptoms, psychosocial impact, and overall burden) that can be tracked over time The single most problematic behavior on TAND-SQ for each participant will be identified by the caregiver and the change over time in that behavior will be assessed using an 11-point Likert-type scale Changes in the TAND clusters (scholastic, neuropsychological, ASD-like, dysregulated behavior, overactive/impulsive, mood/anxiety, eat/sleep) and overall NRS TAND-related symptoms will also be evaluated in the study	Change from baseline at weeks 13, 26, and 52	Under validation as part of the TANDem project [21]	20–30 min
CBCL [33,34]/ABCL [35,36] Caregiver-reported questionnaires that examine internalizing behaviors (e.g., depression and anxiety), externalizing behaviors (e.g., aggression), stress, obsessive-compulsive behaviors, and “sluggish cognitive tempo” These questionnaires allow for an assessment of the participant’s behaviors on a 3-point Likert scale	Change from baseline at weeks 26 and 52	CBCL, ABCL, and ASR are components of ASEBA, a widely used and validated measure to assess behavioral, emotional, and social problems and adaptive functioning in individuals of all ages [37] Versions of the CBCL are validated in participants aged 1.5–5 years and 6–18 years CBCL was found to not be as effective as the TAND checklist in detecting neuropsychiatric and psychopathological problems associated with TSC [34] ABCL is validated in participants aged 18–59 years; however, in the current study, it will be used for participants aged 18–65 years	15–20 min
ASR^a^ [36] The ASR is a participant-reported version of the ABCL	Change from baseline at weeks 26 and 52	ASR is validated in participants aged 18–59 years; however, in the current study, it will be used for participants aged 18–65 years	15–20 min
PROMIS [38–40] Developed by the NIH as a collection of standardized outcome assessments for use in clinical studies This study will incorporate caregiver-assessed short-form PROMIS scales pertaining to the following domains: emotional distress (anger/irritability, anxiety), cognition, positive affect, and sleep disturbance	Change from baseline to weeks 26 and 52 in PROMIS domains	Can be used for children and adults Widely used and validated in large clinical trials [40]	≈5 min/questionnaire
BRIEF [41,42] A set of questionnaires to evaluate executive function from multiple perspectives Theoretically and statistically derived scales measure behavioral aspects such as the ability to control impulses, move freely between situations, modulate responses, anticipate future events, and monitor how an individual’s behavior impacts others BRIEF questionnaire will be completed by the caregiver	Change from baseline at week 26	BRIEF-P: validated for use in participants aged 2–5 years [43] BRIEF2: validated for use in individuals aged 6–18 years [44] BRIEF-A: validated for use in individuals aged >18 years [45] Questionnaires have been used in epilepsy studies and can be used with children who have developmental or acquired neurologic conditions such as learning disabilities, attention disorders, traumatic brain injury, lead exposure, pervasive developmental disorders, depression, and other developmental, neurologic, psychiatric, and medical conditions	10–15 min/ questionnaire
Abbreviated CSHQ [46,47] A caregiver-reported tool used to assess sleep habits and possible difficulties with sleep during the past week Includes items regarding bedtime habits, sleep behaviors, waking through the night, and morning wake-up	Change from baseline at week 26	Well-established sleep measure Validated in children 4–10 years and 2.5–5 years; has been used in children up to 12 years of age [48]	≈10 min
PSQI^a^ [49,50] A participant-reported tool that measures overall sleep quality across the following domains: subjective sleep quality, sleep latency, sleep duration, habitual sleep efficiency, sleep disturbances, use of sleep medicine, and daytime dysfunction during the past month Includes additional questions for the participant’s bed partner or roommate that are qualitatively assessed and do not contribute to the overall score	Change from baseline at week 26	Widely used screening tool for sleep dysfunction in nonclinical and clinical settings [51] Reliable and validated for use in individuals ≥18 years [51]	≈10 min
PedsQL [52] A questionnaire (caregiver-reported in this study) that assesses a participant’s QoL across physical, emotional, and social functioning, as well as functioning at school	Change from baseline to week 26	The PedsQL can be completed by children and young people, with versions available for people aged 5–7, 8–12, and 13–18 years; parent-rated versions are available for children aged 2–4, 5–7, 8–12, and 13–18 years It has been shown to be internally consistent and can distinguish between healthy children and those with health problems [53]	≈5 min
PedsQL FIM [54] A caregiver-reported scale that assesses the impact of pediatric chronic health conditions on parents/ guardians and the family Includes subscales that assess the impact on family daily activities and family relationships, as well as the parents’/guardians’ self-reported physical, emotional, social, and cognitive functioning, communication, and worry	Change from baseline to week 26	Preliminary reliability and validity demonstrated in families with children with complex health conditions [54] Shown to be a reliable and valid measure within a community sample [55]	≈10 min
**Seizure Outcomes**			
The proportion of participants with ≥25%, ≥ 50%, ≥ 75%, and 100% reduction (responder rates) from baseline The proportion of participants experiencing a worsening, no change, or improvement in seizure frequency from baseline	Change from baseline in the proportion of participants with a response at each evaluable visit	—	—
Seizure-free days	Change from baseline in the number of seizure-free days at weeks 4, 13, 26, and 52		
**Other Outcomes**			
CGI-S [56], CareGI-S [57]/PGI-S^b^ [58] The CGI-S and PGI-S/CareGI-S are single-item, 5-point Likert-type scales (ranging from “none” to “very severe”) that are widely used in clinical studies to assess severity of illness Using this scale, clinicians and participants/caregivers will rate their impression of the participant’s problems with behaviors and seizures	Change from baseline at weeks 4, 13, 26, and 52	Can be used for all age groups The CGI rating scales are commonly used clinician-rated measures of global symptom severity and treatment response for patients with mental disorders, but many researchers consider them to be subjective because they involve making judgments based on the clinician’s experience [59] Single-item global impression of change scales have also been used extensively in clinical trials to obtain patients’ and caregivers’ impressions of change in the condition of patients [60]	≈5 min
Treatment retention Ratio between the number of participants continuing treatment at a specific visit over the total number of participants	Retention at weeks 13, 26, and 52 after treatment initiation	–	–

^a^Completion of these participant-reported outcomes is optional in this study.

^b^The CareGI-S is required in this study, whereas the PGI-S is optional.

ABC, Aberrant Behavior Checklist; ABCL, Adult Behavior Checklist; ASD, autism spectrum disorder; ASEBA, Achenbach System of Empirically Based Assessment; ASR, Adult Self Report; BRIEF, Behavior Rating Inventory of Executive Function; BRIEF2, BRIEF Second Edition; BRIEF-A, BRIEF for Adults; BRIEF-P, BRIEF Preschool; CareGI-S, Caregiver Global Impression of Severity; CBCL, Child Behavior Checklist; CGI-S, Clinician Global Impression of Severity; CSHQ, Children’s Sleep Habits Questionnaire; NIH, National Institutes of Health; NRS: numeric rating scale; PedsQL, Pediatric Quality of Life Inventory; PedsQL FIM, PedsQL Family Impact Module; PGI-S, Patient Global Impression of Severity; PROMIS, Patient-Reported Outcomes Measurement Information System; PSQI, Pittsburgh Sleep Quality Index; QoL, quality of life; TAND, TSC-associated neuropsychiatric disorders; TAND-SQ, TSC-Associated Neuropsychiatric Disorders Self-Report, Quantified Checklist; TSC, tuberous sclerosis complex.

### TAND outcomes

TAND-specific outcomes will be evaluated using TAND-SQ, which was derived from the TAND Checklist Lifetime Version [20] and is currently being validated by the Global TANDem Consortium [19]. The caregivers will identify the single most problematic behavior and track changes over time through their responses in the TAND-SQ. The trial is designed to assess changes in TAND-associated outcomes that are of importance to the caregivers and individuals with TSC; therefore, the most problematic behavior score is being used to evaluate the caregiver’s impression of change in the problem that is most bothersome to them or the participant, regardless of the severity. Evaluation of the most bothersome symptom has been conducted previously and is recommended by the U.S. Food and Drug Administration for patient-focused drug development [61–63]. Natural TAND clusters—scholastic, neuropsychological, autism spectrum disorder-like, dysregulated behavior, overactive/impulsive, mood/anxiety, and eating/sleeping [22]—will be used to group specific neuropsychiatric characteristics for analysis. Absolute and change from baseline in TAND cluster scores, as measured by average numeric rating scale (NRS), and overall NRS TAND-related symptoms will be summarized at weeks 13, 26, and 52 by using standard descriptive statistics for continuous variables.

Given that the TAND-SQ is a relatively new instrument for assessment of outcomes specific to TSC, it will be complemented by additional well-established tools, such as Aberrant Behavior Checklist [26–32], which caregivers will use to rate the participant’s specific symptoms across the following subscales: irritability, social withdrawal, stereotypic behavior, hyperactive noncompliance, and inappropriate speech. Child Behavior Checklist (CBCL) [33,34], and Adult Behavior Checklist (ABCL) [35,36] or Adult Self Report (ASR) [36] will also be used, as applicable, on the basis of the participant’s age and ability. CBCL, ABCL, and ASR are components of the Achenbach System of Empirically Based Assessment, a validated measure to assess behavioral, emotional, and social problems and adaptive functioning across ages [37]. Changes in the overall severity of behavioral symptoms will be assessed by the caregivers/participants using the CareGI-S/Patient Global Impression of Severity (PGI-S) scale [58] and by clinicians using the Clinician Global Impression of Severity (CGI-S) scale [57,59,60].

To assess effects on specific TAND domains, additional instruments will be included, such as the Patient-Reported Outcomes Measurement Information System (PROMIS), which uses latest advances in information technology, psychometrics, and health survey research to measure patient-reported outcomes, because of the increasing focus on moving toward the use of patient-centered assessment tools [38–40]; executive functioning outcomes evaluated using Behavior Rating Inventory of Executive Function (BRIEF) [41–43,45]; and sleep outcomes evaluated using Children’s Sleep Habits Questionnaire (CSHQ) [46–48] or Pittsburgh Sleep Quality Index (PSQI) [49–51]. Additionally, Pediatric Quality of Life Inventory (PedsQL) [52,53] and the PedsQL Family Impact Module (PedsQL FIM) [54,55] will be used to evaluate QoL and family functioning.

The analysis of TAND-related outcomes will be done in participants with and those without seizure responses to CBD treatment using TAND-SQ, CBCL/ABCL/ASR, BRIEF, PROMIS, CGI-S, and PGI-S/CareGI-S to explore the TAND outcomes independent of the effect of CBD treatment on seizure outcomes.

### Seizure-related outcomes

Seizure-related outcomes will include treatment responder rates (participants with ≥25%, ≥50%, ≥75%, and 100% reduction in seizure frequency from baseline). The proportion of participants who experience a worsening, no change, or improvement in seizure frequency will be evaluated, along with the change in the number of seizure-free days from baseline. Change in overall severity of seizure symptoms will be evaluated by caregivers/participants using the CareGI-S/PGI-S scale and by clinicians using the CGI-S scale.

Participant retention at weeks 13, 26, and 52 following the initiation of treatment will be evaluated.

### Safety and tolerability

Safety and tolerability assessments will include incidence and severity of treatment-emergent adverse events (AEs), incidence of serious AEs, discontinuations because of AEs, number of inpatient hospitalizations due to epilepsy, abnormal clinical laboratory parameters, suicidal ideation, and behavior risk monitoring throughout the study duration. At the end of the first treatment period, caregivers will be given a short questionnaire of approximately three items to determine reasons for remaining on or discontinuing CBD treatment.

### Statistical analysis

EpiCom is an exploratory study, so no formal statistical hypothesis will be tested. Ranking is not assigned to the study endpoints; therefore, no hierarchical testing strategy or other multiplicity adjustments will be considered. Given that the study is not controlled, no direct causal attribution of any observed association to CBD treatment will be made based on study findings. The study is not designed to distinguish whether improvements in TAND outcomes are due to CBD treatment or if they are the result of seizure reduction. A modelling approach will be used to explore the extent to which the observed associations of CBD treatment with TAND endpoints is associated with the change in seizure frequency during follow-up. Its marginal/causal effects may be further investigated with an exploratory mediation analysis [64].

Outcomes will be reported using standard descriptive statistics for continuous variables, including 95% CIs for the mean (including SD) or median (including the first and third quartiles, and the minimum and maximum values) change. Counts and percentages will be reported along with the 95% CI of the rate. Statistics will be presented overall and by age, where applicable. If provided, *P* values will be declared as nominal. An intermediate analysis at week 26 will be performed to describe any early trends.

A generalized linear mixed model (GLMM) will be used to evaluate the significant change from baseline to week 26 in effectiveness endpoints, adjusted by the assessment at baseline and age at baseline. During the second treatment period, summary statistics for endpoints may be computed for and between the CBD treatment and SoC arms. At week 52, a GLMM will be used to describe the association of treatment with change from baseline in effectiveness endpoints, adjusted by assessment at baseline, and a propensity score calculated based on age at baseline, sex, percentage change from baseline at week 26 in seizure frequency, 28-day average seizure-free days at week 26, and the number of concomitant ASMs. Least-squares means of the treatment effect (CBD vs SoC), two-tailed 95% CI and the unadjusted *P* values will be generated if at least 25% of the participants reaching the 26-week interim review will opt for SoC treatment in the second treatment period.

All participants who sign the informed consent form and take ≥1 dose of CBD will be included in analysis of safety endpoints (safety analysis set). Participants who, in addition to the above criteria, also complete ≥1 postbaseline assessment will be included in the effectiveness endpoints analysis (full analysis set). The raw data captured electronically will be converted to analysis datasets according to the Clinical Data Interchange Standards Consortium standards and principles. Data management and analyses will be performed using SAS version 9.4 (or higher) of the SAS Institute.

### Sample size

Planned enrollment is for 75 participants, such that 60 participants may be evaluable at the 26-week interim review (assuming a 20% withdrawal rate during the first treatment period). Participants will be considered evaluable for a given endpoint if they complete the endpoint assessment at baseline and at the 26-week interim review. Although not required, the study will attempt to enroll approximately 25 adult participants.

### Enrollment

Participants will be primarily recruited from TSC specialist clinics globally. The study will be conducted at approximately 20 sites across the US, the UK, Canada, and Poland (list of study sites available on https://clinicaltrials.gov). The study is estimated to run from June 2023 to January 2026, with intermediate analyses planned when patients have completed three or six months of study intervention.

### Ethical considerations

The study will be conducted in accordance with the consensus ethical principles derived from international guidelines including the Declaration of Helsinki and Council for International Organizations of Medical Sciences International Ethical Guidelines, International Council for Harmonisation Good Clinical Practice Guidelines, and the applicable laws and regulations of the participating countries. The protocol and amendments were approved by institutional review board or independent ethics committee and national regulatory authority (as applicable) before initiation. The protocol was approved by WCG Institutional Review Board in the United States; received favorable opinion from South Central – Oxford A Research Ethics Committee; and was approved by the Health Research Authority and Health and Care Research Wales in the UK. The protocol has also been approved by the European Union Clinical Trials Information System for Poland. Written informed consent will be obtained from all participants or the participants’ parent(s)/legally authorized representatives prior to any study-related procedures.

## Discussion

There is an increasing focus on incorporation of “patient experience data” into decision-making for clinical trials and drug development process [63]. Including patient experience information in trial design gives patients a voice and allows for evaluation of endpoints that are of importance to them and their families. The EpiCom study was designed to increase the potential for efficient study execution, data dissemination, and impact on patient care.

In addition to keeping patient and caregiver input at the center of the study design process, EpiCom aims to reduce the burden associated with trial participation by implementing a hybrid decentralized clinical trial (DCT) approach, in which participants will have the choice to attend the clinical trial site, conduct the assessments at home, or both. The DCT strategy allows for clinical trial activities to be conducted at locations other than traditional clinical trial sites, such as the patients’ homes or other locations remote from the clinical trial site (e.g., access to video call visits, home phlebotomy for blood collection, and home delivery of study drug), as well as the use of digital health technologies (e.g., access to assessment tools via their own mobile device or directly on the web). Using the DCT approach, more participants can access the investigational treatment, which in turn can support inclusion of a greater diversity of patient populations and increase efficiency by reducing the burden on caregivers and enhancing engagement among participants, thereby increasing patient recruitment and retention [65].

Highly purified CBD (Epidiolex^®^) is already an approved treatment for seizures associated with TSC in many countries; therefore, because of ethical considerations and based on advice from PAGs, EpiCom was designed as an open-label, single-arm trial to ensure that CBD is available to all eligible participants. The assessment tools were selected primarily on the basis of what the advisory panel members considered the most effective method for measuring outcomes that are of value to the participants of the trial. Other considerations were the validation status of the tools, their previous use in TSC or similar studies, and the time needed to complete the assessments and their complexity.

Importantly, both the TAND-SQ and the TSC-PROM are condition-specific measures and were developed in direct participation with a broad range of stakeholders from the TSC community. The TAND-SQ development, for example, included family members as active members of the TAND consortium throughout the process of development and validation of the tool [19,23]. Given that TSC is associated with a wide range of symptoms and affects multiple organs in the body, a “holistic” approach has been proposed to manage TSC and TAND-associated symptoms [6,66], with an emphasis on a systematic strategy to study and manage TAND symptoms, involving standardized approaches toward behavioral assessments and neuropsychological evaluations [4,6].

Collaborating with the TSC community enables focus on the clinical study objectives that are most important to the community, thus providing relevance to the study results, with the hope of affecting and adding value to patient care. The collaborative approach implemented for this study also increases the potential for efficient study execution and data dissemination. The option to withdraw from CBD treatment while still maintaining the SoC at 26 weeks may also improve patient/caregiver engagement, recruitment, and retention.

Beyond the questionnaires, we believe qualitative interviews may further improve and enrich the analysis of collected data. In the absence of group interviews, which had to be abandoned for logistical reasons, the caregivers are interviewed at 26 weeks using a short questionnaire that includes three free-text items to capture reasons for continuing or discontinuing CBD. This approach allows individual qualitative experiential findings to be linked to quantitative data. While the absence of group interviews represents a missed opportunity, qualitative group interviews should be incorporated as an integral part of future research in this area. Qualitative interviews during the study may further improve and enrich the collected data.

Given the multicountry nature of the study, descriptive data on patient characteristics by country may be evaluated as a post hoc analysis. In addition, the real-world nature of the study allows for any analysis on participant subgroup receiving concomitant medications to understand overall impact of these medication with CBD and TAND.

A limitation of the study is lack of a specific statistical strategy to measure the response bias and bias recalibration (change in response bias between two time periods). To mitigate this, the study’s statistical analysis uses a GLMM on repeated endpoints to quantify the magnitude of the dimension scale score’s difference from the baseline. The model accounts for within-subject correlation, confounding factors, and baseline assessments to reduce variability and the presence of reporting bias. Additionally, caregivers may have completely different views on the “severity-rating” of certain behaviors experienced. Such a subjective symptom under any circumstance would be challenging to standardize. To increase data accuracy, consistency and impartiality, several different questionnaires were included in the protocol to assess changes in the same nonseizure outcomes, including the patient’s most problematic behavioral symptom from clinician and caregiver perspectives. Also, to reduce intra- and interparticipant variability, training materials such as a behavioral severity discussion guide were designed in collaboration with PAGs to support discussions between study investigators and caregivers (who will be administering the assessments), to align on severity grading of the participant’s behavioral symptoms prior to formal consent and screening procedures.

We are continuing to work in partnership with an engaged and integrated patient/caregiver-HCP community that is enthusiastic and invested in the support of the clinical study delivery and interpretation. The use of the collaborative approach described here could serve as a blueprint to inform future studies evaluating potential pharmacotherapies for neuropsychiatric outcomes in TSC and similar populations. This study began enrolling participants from the US in June 2023. The UK, Canada, and Poland will soon follow. Last patient in is expected in the first quarter of 2025 with database lock in first quarter of 2026.

## Supporting information

S1 Table Summary of key insights and recommendations from advisory boards. (PDF)

S1 Checklist SPIRIT checklist. (PDF)
