# Supplementary material for: Protocol for EpiCom: A phase 3b/4 study of behavioral outcomes following adjunctive cannabidiol for the management of tuberous sclerosis complex-associated neuropsychiatric disorders (TAND)
Source: PLoS One. 2025 Jun 12;20(6):e0324648. doi: 10.1371/journal.pone.0324648 (PMC12161588; doi:10.1371/journal.pone.0324648)
Supplement: S1 Table — (PDF) [file pone.0324648.s001.pdf]

Supporting material for:

Protocol for EpiCom: a phase 3b/4 study of behavioral outcomes following adjunctive cannabidiol for the management of tuberous sclerosis complex-associated neuropsychiatric disorders (TAND)

van Eeghen AM, et al.

S1 Table. Summary of Key Insights and Recommendations From Advisory Boards

	Patients and Caregivers	HCPs
**Study Objectives**		
Behavior	Reduction of seizures has a direct impact on behavior	Measures are rating scales: trial focus – behavioral, not direct neuropsychological functioning
Executive function	Executive function affects day-to-day independence and has a major impact on family life	Executive skills are commonly affected in TSC
Sleep	Sleep influences everything. All measures are impacted by sleep for individuals and caregivers	Sleep is critical for assessing the impact on primary outcome measures
Mood	Mood is a consequence of other factors and is unlikely to need a separate measure	Behavior and mood not defined. Align with TAND language when there are seven distinct clusters
**Study Design and Delivery**		
Study duration	Reasonable study length with good study communication. Patients and caregivers are happy to help design the study	Two-step design: explore short-term gains, then refine subgroups for longer time period
Use of placebo	The 50:50 gamble (of placebo) is a barrier for families joining studies	Difficult to have a balanced control group because of natural TSC variability
Recruitment/ retention	Virtual visits: remove a barrier to entry; provide better insight into people's behavior when they are in their own environment vs at study site	Preventing change to other medications increases dropout. People rarely stay stable for 6 months to 1 year
Clinic vs. home	Clinic visits must be flexible and efficient because they have a direct influence on observed behavior	Completion of scales by caregivers at home increases variability. Better to assess under standard conditions
**Outcome Measures**		
Outcome measurement	Previous experience of measures shared	Beneficial to use tools used in clinical practice setting

Selection considerations	Reflections on utility, scoring systems, and specific tools. Recommend TAND to profile each individual, then subgroup	TAND: developing a systematic way to study co-occurring effects of CBD that are beneficial
	TAND-SQ (self-quantified) is being validated	Reflections on focused tools used in clinic/research, time, complexity, improvements being released (e.g., BRIEF, CBCL/ABCL, BASC, PROMIS)
	Executive function focus: impact on daily living tasks	A clinically meaningful definition of outcome needed
	Use devices to accurately record sleep	
	Previous experience of measures shared	
**Study Population**		
Personalization	Everyone with TSC has individual experience	Universal primary objective, then cluster into personalized subgroups
Eligibility criteria	IQ exclusion not pragmatic: group by communication ability	IQ <60 should not be an exclusion criterion
	Include all seizure severities; those with milder seizures can communicate feelings better	Careful review of seizure type, frequency, and severity
	Suicidal ideation should not be excluded. It is a part of TSC and a fact of life	Exclusion of suicidal behavior is an FDA mandate, but it biases and limits sample; therefore, remove
	About 50% of patients are on the [autism] spectrum	Potential for birth control requirement
	IQ exclusion not pragmatic: group by communication ability	Careful consideration for everolimus use
		Washout period for previous artisanal CBD would be needed at start

ABCL, Adult Behavior Checklist; BASC, Behavior Assessment System for Children; BRIEF, Behavior Rating Inventory of Executive Function; CBCL, Child Behavior Checklist; CBD, cannabidiol; FDA, US Food and Drug Administration; HCPs, healthcare professionals; PROMIS, Patient-Reported Outcomes Measurement Information System; TAND, TSC-associated neuropsychiatric disorders; TAND-SQ, TAND Self-Report, Quantified Checklist; TSC, tuberous sclerosis complex.
